# Supplementary material for: Constraining chemical transport PM2.5 modeling outputs using surface monitor measurements and satellite retrievals: application over the San Joaquin Valley
Source: Atmos Chem Phys. Author manuscript; Available in PMC 2019 Jul 9. (PMC6166888; doi:10.5194/acp-18-12891-2018)
Supplement: Supplement1 [file NIHMS1506411-supplement-Supplement1.pdf]

Supplement of Atmos. Chem. Phys., 18, 12891–12913, 2018
https://doi.org/10.5194/acp-18-12891-2018-supplement
© Author(s) 2018. This work is distributed under
the Creative Commons Attribution 4.0 License.

Supplement of

Constraining chemical transport $PM_{2.5}$ modeling outputs using surface monitor measurements and satellite retrievals: application over the San Joaquin Valley

Mariel D. Friberg et al.

Correspondence to: Mariel D. Friberg (mariel.d.friberg@nasa.gov)

The copyright of individual parts of the supplement might differ from the CC BY 4.0 License.

S1 MISR-RA Retrieval Process

This subsection provides a brief summary of Limbacher and Kahn (2014; 2017). The basic principle of the MISR-RA involves comparing the observed MISR top-of-atmosphere (TOA) reflectances with a pre-built look-up-table (LUT) of simulated TOA reflectances (analogous to a scaled, unitless radiance), and selecting only the aerosol optical depths and mixtures that meet certain goodness-of-fit criteria. To match the MISR-observed TOA reflectances ($\rho_{\lambda,c}^{TOA}$) in four spectral bands (λ) and each of up to nine cameras (c), simulated atmospheric and surface contributions to the TOA reflectance are calculated for a range of possible conditions and tested against the observations. The modeled portion of reflected light that reaches the instrument without interacting with the surface is the path reflectance ($\rho_{\lambda,c}^{path}$), and the modeled portion of reflected light that interacts with the surface is designated $\rho_{\lambda,c}^{surf}$. The MISR-RA uses a single minimization parameter (*M*) to self-consistently retrieve aerosol amount and type, as well as surface reflectance. For any given aerosol optical depth (AOD, or τ) and aerosol mixture combination, the minimization parameter *M* can be represented as:

$$M(\tau) = \sum_{\lambda}\sum_{c} \frac{w_{\lambda,c} * \left[\rho_{\lambda,c}^{TOA} - (\rho_{\lambda,c}^{path} + \rho_{\lambda,c}^{surf})\right]^2}{Unc_{\lambda,c}^2 * \left[\sum_{\lambda}\sum_{c} w_{\lambda,c}\right]} \tag{S1}$$

The channel-specific weights are $w_{\lambda,c}$, and the assumed uncertainty of the entire model/measurement system is $Unc_{\lambda,c}$.

Because $\rho_{\lambda,c}^{surf}$ is not known a priori, this term must be determined before *M* can be computed. This two-step process involves first invoking the principle of angular shape similarity to compute a representation of the surface, by assuming that the angular shape of the Bi-directional Reflectance Distribution Function (BRDF) is fixed, but allowing it to vary spectrally(Diner et al., 2005;North et al., 1999). Applying this additional constraint, the $\rho_{\lambda,c}^{surf}$ term of Eq. (S1) is expanded into $\rho_{\lambda,c}^{surf} = E_{\lambda}^{BOA} * A_{\lambda} * T_{\lambda,c}^{up}$, where E_{λ}^{BOA} is the bottom-of-atmosphere (BOA) downward-directed irradiant reflectance, A_{λ} is the spectral albedo, and $T_{\lambda,c}^{up}$ is the azimuthally integrated transmittance from BOA to the MISR camera of interest. Because A_{λ} is not a function of view angle, we compute the optimal A_{λ} analytically for each band/AOD/mixture combination by taking the derivative of (S1) with respect to A_{λ} and setting this equal to zero, yielding:

$$A_{\lambda} = \frac{\sum_c \left[\frac{w_{\lambda,c}}{Unc_{\lambda,c}^2} * T_{\lambda,c}^{up} * \left(\rho_{\lambda,c}^{TOA} - \rho_{\lambda,c}^{path}\right)\right]}{E_{\lambda}^{BOA} * \sum_c \left[\frac{w_{\lambda,c}}{Unc_{\lambda,c}^2} * \left(T_{\lambda,c}^{up}\right)^2\right]} \tag{S2}$$

The second step of this process requires that we modify $\rho_{\lambda,c}^{surf}$ such that $\rho_{\lambda,c}^{surf} = E_{\lambda}^{BOA} * A_{\lambda} * L_c * T_{\lambda,c}^{up}$, where L_c is a normalized, spectrally invariant but angularly varying, modulation of the surface albedo. This approximation simply implies that although the brightness of the surface can change with view angle, its color does not. Because A_{λ} and L_c cannot be calculated simultaneously, we instead use (S2) to calculate a first guess for A_{λ}, and then take the first derivative of (S1) with respect to L_c, setting it equal to 0, and calculating L_c as follows:

$$L_c = \frac{\sum_\lambda \left[\frac{w_{\lambda,c}}{Unc_{\lambda,c}^2} * E_\lambda^{BOA} * A_\lambda * T_{\lambda,c}^{up} * \left(\rho_{\lambda,c}^{TOA} - \rho_{\lambda,c}^{path} \right) \right]}{\sum_\lambda \left[\frac{w_{\lambda,c}}{Unc_{\lambda,c}^2} * \left(E_\lambda^{BOA} * A_\lambda * T_{\lambda,c}^{up} \right)^2 \right]} \quad \text{(S8)}$$

Substituting A_λ, L_c, and $\rho_{\lambda,c}^{surf} = E_\lambda^{BOA} * A_\lambda * L_c * T_{\lambda,c}^{up}$ into (S1) yields the minimization parameter M for a particular AOD and aerosol mixture over land. (Note that L_c represents the angular dependence of the surface BRDF at the specific MISR view-angles.) The algorithm then selects the best fitting AOD for each of the 774 aerosol mixtures described in Limbacher and Kahn (2014), and saves the AOD, surface albedo, and associated goodness-of-fit parameter (M) for each mixture.

S2 Applying the MISR-RA Retrieval Results to Constraining the Air Quality Model

For comparison with the CTM, we compute aggregate AOD, Angstrom Exponent (ANG), absorption aerosol optical depth (AAOD), and non-spherical aerosol optical depth values from the RA results. As described below, we also compute aggregate AOD retrieved for the spherical absorbing aerosol components, and separately for spherical non-absorbing aerosol components. These aggregated parameters are calculated by weighting the respective parameters for each passing mixture by 1/M, such that better fitting mixtures are weighted more heavily than poorer fitting ones. The threshold value of M used to determine passing mixtures is set to 1.25 * M_{min} + 0.25(Limbacher and Kahn, 2014). Because aerosol retrievals are affected by a range of conditions such as solar and viewing geometry, surface brightness, AOD, and aerosol type, we highlight below some of the key factors that help determine aerosol retrieval sensitivity (Kahn and Gaitley, 2015).

- *Surface brightness* – As the surface becomes brighter, the algorithm loses some sensitivity to all retrieved aerosol properties (including AOD). This occurs because, other things being equal, the contribution of $\rho_{\lambda,c}^{path}$ relative to $\rho_{\lambda,c}^{surf}$ decreases as surface brightness increases.
- *Number of cameras used* – To separate the two terms, the RA uses the property that $\rho_{\lambda,c}^{path}$ generally increases at steeper view angles, whereas $\rho_{\lambda,c}^{surf}$ generally decreases at steeper view angles. This also means that the number of cameras used can influence retrieval sensitivity to AOD and aerosol type.
- *Scattering Angle Range* - Other things being equal, a greater range of angles sampled by the MISR cameras relative to the solar direction offers higher confidence in the retrieved particle properties. As the aerosol scattering phase function peaks in the forward direction, retrieval sensitivity also tends to increase as the minimum scattering angle sampled decreases.
- *Retrieved Aerosol Optical Depth* - Mixture sensitivity diminishes when AOD is below about 0.15 or 0.2, although this also depends on other retrieval conditions.

S3 Constrained CMAQ Simulation Using Ground-based Observations

Ambient ground observations are used to constrain CTM model simulations (i.e., CMAQ), to estimate daily, spatially refined pollutant metrics and associated correlations. The method provides daily spatial field estimates of air pollutant concentrations and uncertainties that are consistent with observations at the ground stations, as well as with assumed emissions and modeled meteorology; it is summarized briefly below, and is described in detail by Friberg et al. (2016).

The constrained spatiotemporal concentration "fused" dataset (C_{FCMAQ}) is built using weighted fields of daily interpolated surface observation ratios and daily adjusted CMAQ result ratios that are rescaled to the estimated two-month study period mean fields (Eq. S4):

$$C_{FCMAQ_{s,t}} = \alpha \overline{C_{CMAQ_s}}^{\beta} \left[W_{s,t} \left\{ \frac{C_{SURF_{s_m,t}}}{\overline{C_{SURF_{s_m}}}} \right\}_{krig} + \left(1 - W_{s,t}\right) \left\{ \frac{C_{CMAQ_{s,t}}}{\overline{C_{CMAQ_s}}} \right\} \right] \tag{S4}$$

Here, α is a regression parameter that adjusts the amplitude to account for inter-monthly differences, C_{CMAQ} represents CMAQ simulated concentrations, the overbar indicates two-month temporal averaging, β is assumed to be a constant, species-specific regression parameter that accounts for interspecies nonlinearity differences, s indicates spatial location, t represents time, W is a weighting factor, C_{SURF} represents observed concentrations, and s_m indicates monitor locations. Neither inter-monthly nor seasonal corrections were applied. Scaling the daily ratio fields by the spatially regressed two-month mean observations reduces model biases. The estimated mean fields are developed from CMAQ-derived mean spatial fields adjusted to observed means using power regression models for the two-month time period of the current study. These regression parameters are species-specific, because CMAQ biases differ among the PM species.

The daily-resolved, observation-based ratio fields capture the robust temporal variance characterized by ground monitors. These concentration fields are calculated by spatially interpolating the normalized, daily-observed concentrations using kriging. As shown by Friberg et al. (2017), the daily-adjusted CMAQ result ratios capture the spatial variance while reducing bias. The optimization is based on a spatiotemporal weighting factor (W) that maximizes the degree to which the observation-based estimate captures temporal variation relative to the CMAQ-based estimate, as a function of distance from the observation (Eq. S5). Due to missing data, the weighting factors vary over time as well as space. The temporal Pearson correlation fields of the daily observation-based fields, R_1, are derived using an exponential correlogram modeled to fit the isotropic spatial autocorrelation of the observations (Eq. S6). The fitted parameters include the intercept that results from instrument error, estimated by collocated instruments (R_{coll}), the distance from a grid centroid to the nearest observation on a given day (x), and the range (r) at which the correlation between monitors has decreased to an e-folding value of R_{coll}. The term R_1 varies over space and time because the observation frequency varies among monitors. The average of the temporal correlations between the CMAQ simulations and observations across all monitors (n_{s_m}) is used to estimate R_2, which represents the estimated temporal correlation of the daily adjusted CMAQ results ratio fields and ambient pollution (Eq. S7).

The spatiotemporal weighting factor is also applied to the observation- and CMAQ-based temporal correlation fields to quantify the uncertainties of the optimized spatiotemporal concentration dataset (R_{FCMAQ}; Eq. S8).

$$W_{s,t} = \frac{R_{1_{s,t}}(1-R_2)}{R_{1_{s,t}}(1-R_2)+R_2\left(1-R_{1_{s,t}}\right)} \qquad \text{(S5)}$$

$$R_{1_{s,t}} \approx R_{coll} e^{-x_{s,t}/r} \qquad \text{(S6)}$$

$$R_2 \approx \frac{1}{n_{s_m}} \sum\nolimits_{s_m} corr\left(C_{SURF_{s_m}}(t), C_{CMAQ_{s_m}}(t)\right) \qquad \text{(S7)}$$

$$R_{FCMAQ_{s,t}} = \begin{cases} W_{s,t} R_{1_{s,t}} + \left(1 - W_{s,t}\right) R_2 & R_1 > R_2 \\ R_2 & R_1 =< R_2 \end{cases} \qquad \text{(S8)}$$

Table S1: Aerosol groupings and definitions used in the PM2.5 mass reconstruction for EPA CSN and CMAQ v5.0.2 output species.

Aerosol Groups	Aerosols	CSN Monitor Representation	CMAQ Species Representation [1]
Inorganic Ions (II)	Sulfate	SO4	ASO4I+ASO4J+ASO4K
	Nitrate	NO3	ANO3I+ANO3J+ANO3K
	Ammonium	NH4	ANH4I+ANH4J+ANH4K
Light absorbing Carbon (LAC)	Elemental Carbon (EC)	EC	AECI+AECJ
Organic Matter (OM)	Organic Carbon (OC)	1.8 x OC	AALKJ+ABNZ1J+ABNZ2J+ABNZ3J+AISO1J+AISO2J+AISO3J+AOLGAJ +AOLGBJ+AORGCJ+ASQTJ+ATOL1J+ATOL2J+ATOL3J+ATRP1J+ ATRP2J+AXYL1J+AXYL2J+AXYL3J+(1.4-0.2)*(APOCI+APOCJ)
Sea Salt (SS)	Sea Salt	1.8Cl + 1.4486Na+ 1.63Ca+ 1.2(K-0.6Fe)	ANAI+ANAJ+ACLI+ACLJ+ASEACAT+ACAJ+AKJ+AMGJ
Dust	Dust	2.49Si+1.94Ti +2.42Fe	AOTHRI+AOTHRJ+ACORS+ASOIL+AFEJ+ASIJ+ATIJ+AMNJ+APNCOMI +APNCOMJ

[1] Model species in the Aitken, accumulation, and coarse size modes are indicated by the letters I, J, and K, respectively.

Table S2: Classification of aerosol groups according to MISR-RA microphysical property components.

<table>
<tr><th>Aerosol Group</th><th colspan="2">Light-Absorbing Carbon</th><th colspan="5">Inorganic Ions + Organic</th><th colspan="2">Dust</th></tr>
<tr><td>Component Shape</td><td colspan="7">Spherical</td><td colspan="2">Non-Spherical</td></tr>
<tr><td>Component Absorption</td><td>Strongly Absorbing</td><td>Moderately Absorbing</td><td colspan="5" rowspan="2">Non-Absorbing</td><td colspan="2">Varying Absorption Dust</td></tr>
<tr><td>Component Description</td><td>Smoke Pollution</td><td>Smoke Pollution</td><td>Transported</td><td>Coarse</td></tr>
<tr><td>Component Effective Radius (µm)</td><td>0.12</td><td>0.12</td><td>0.06</td><td>0.12</td><td>0.26</td><td>0.57</td><td>1.28</td><td>0.75</td><td>2.40</td></tr>
<tr><td>Single-Scattering Albedo (558 nm)</td><td>0.82</td><td>0.91</td><td colspan="5">1.00</td><td>0.98</td><td>0.90</td></tr>
</table>

*See Limbacher and Kahn (2014) for detailed component definitions.

Table S3: The empirically based factors of Eq. (11) and their respective literature sources.

Species	Specific dry efficiency factor [m^2 g^{-1}]	Hygroscopic growth factor $f_{RH(z)}$[unitless]=$bscat_{wet}/bscat_{dry}$	Single scattering albedo (ω)
Inorganic Ions	3<br>(Malm et al. 2007; Chow et al. 2015)	varies<br>(Malm et al., 2011; Song et al., 2008)	1
Organic Matter	4<br>(Malm et al. 2007; Chow et al. 2015)	varies<br>(Zamora and Jacobson, 2013)	ω_{OM}=.99<br>(Sun et al.,2007; Bond and Bergstrom, 2006)
Sea Salt	1.37<br>(Malm et al. 2007;Chow et al. 2015)	varies<br>(Park et al., 2014)	ω_{SS}=1
Light Absorbing Carbon	10<br>(Malm et al. 2007; Chow et al. 2015)	0	ω_{LAC}=Temp*0.928 (Conant et al., 2003);<br>α_{abs}=7.5±1.2 [m^2/g^{-1}]<br>(Bond and Bergstrom, 2006)
Dust	stratifying by size (Park et al., 2011);<br>βi ~regressed value (Tegen et al., 1996)	0	regressed value<br>(Tegen et al., 1996)

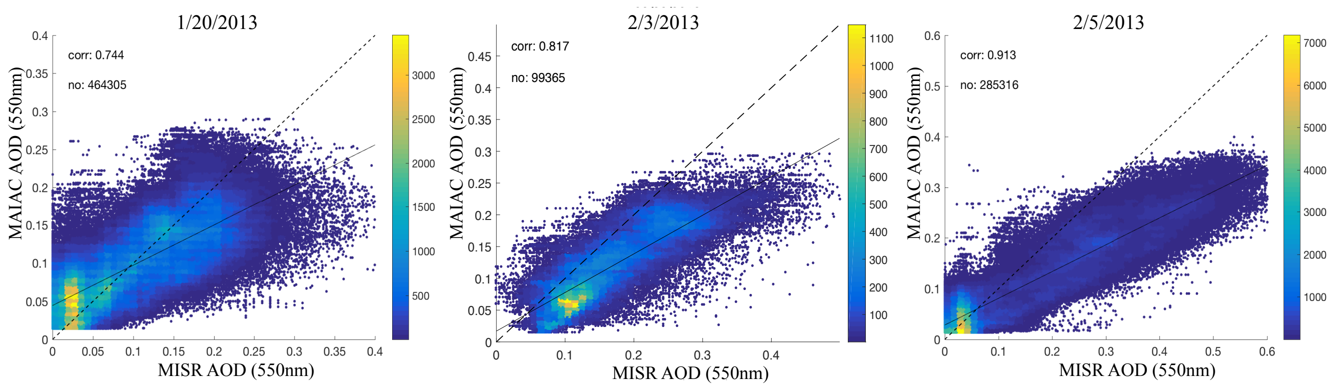

Figure S1: Density scatterplots comparing MISR-RA and MAIAC-Terra retrieved AOD for January 20th, February 3rd, and February 5th. A solid linear regression line and a 1:1 dashed line are shown for reference.

Table S4: Summary Statistics for AERONET Coincidences with MISR-RA and MAIAC within 15 minutes of Terra overpass time, for AOD≥0.15 and (all AOD).

Date	Dataset	No. Coincident Observations	Mean	SD	Spatial R	RMSE	NRMSE	MB	MAE	NMB	NME
1/18/13	MISR	- (1)	- (0.097)	- (-)	- (-)	- (0.017)	- (0.153)	- (-0.017)	- (0.017)	- (-0.153)	- (0.153)
	MAIAC	- (14)	- (0.08)	- (0.022)	- (0.261)	- (0.032)	- (0.314)	- (-0.022)	- (0.027)	- (-0.218)	- (0.267)
	FillSAT	- (1)	- (0.097)	- (-)	- (-)	- (0.019)	- (0.168)	- (-0.019)	- (0.019)	- (-0.168)	- (0.168)
	CMAQ	- (14)	- (0.07)	- (0.032)	- (0.63)	- (0.041)	- (0.398)	- (-0.033)	- (0.035)	- (-0.321)	- (0.344)
1/20/13	MISR	8 (11)	0.191 (0.172)	0.04 (0.048)	0.854 (0.913)	0.021 (0.019)	0.113 (0.113)	0.002 (0.004)	0.015 (0.014)	0.01 (0.021)	0.082 (0.082)
	MAIAC	9 (13)	0.161 (0.138)	0.049 (0.054)	0.334 (0.658)	0.056 (0.052)	0.288 (0.302)	-0.034 (-0.034)	0.04 (0.039)	-0.173 (-0.196)	0.206 (0.226)
	FillSAT	9 (13)	0.197 (0.174)	0.041 (0.05)	0.872 (0.923)	0.022 (0.019)	0.111 (0.109)	0.002 (0.002)	0.017 (0.015)	0.01 (0.01)	0.088 (0.085)
	CMAQ	9 (13)	0.133 (0.107)	0.043 (0.057)	0.625 (0.852)	0.07 (0.072)	0.358 (0.418)	-0.062 (-0.066)	0.063 (0.066)	-0.319 (-0.381)	0.324 (0.385)
2/3/13	MISR	1 (1)	0.242 (0.242)	- (-)	- (-)	0.02 (0.02)	0.078 (0.078)	-0.02 (-0.02)	0.02 (0.02)	-0.078 (-0.078)	0.078 (0.078)
	MAIAC	4 (6)	0.189 (0.167)	0.034 (0.044)	0.854 (0.89)	0.025 (0.021)	0.131 (0.123)	0 (-0.004)	0.025 (0.02)	-0.002 (-0.021)	0.13 (0.116)
	FillSAT	1 (1)	0.242 (0.242)	- (-)	- (-)	0.025 (0.025)	0.093 (0.093)	-0.025 (-0.025)	0.025 (0.025)	-0.093 (-0.093)	0.093 (0.093)
	CMAQ	6 (8)	0.121 (0.101)	0.107 (0.098)	0.971 (0.975)	0.166 (0.151)	0.608 (0.635)	-0.152 (-0.137)	0.152 (0.137)	-0.555 (-0.575)	0.555 (0.575)
2/5/13	MISR	8 (8)	0.377 (0.377)	0.128 (0.128)	0.99 (0.99)	0.045 (0.045)	0.134 (0.134)	0.04 (0.04)	0.04 (0.04)	0.119 (0.119)	0.119 (0.119)
	MAIAC	8 (9)	0.23 (0.207)	0.077 (0.1)	0.914 (0.927)	0.1 (0.1)	0.316 (0.338)	-0.088 (-0.089)	0.09 (0.091)	-0.277 (-0.302)	0.283 (0.307)
	FillSAT	11 (12)	0.37 (0.35)	0.114 (0.13)	0.977 (0.982)	0.04 (0.038)	0.117 (0.119)	0.032 (0.03)	0.032 (0.03)	0.096 (0.093)	0.096 (0.093)
	CMAQ	11 (12)	0.154 (0.151)	0.048 (0.047)	0.257 (0.31)	0.212 (0.203)	0.628 (0.635)	-0.185 (-0.169)	0.185 (0.169)	-0.546 (-0.528)	0.546 (0.529)
2/12/13	MISR	1 (8)	0.121 (0.107)	- (0.017)	- (0.392)	0.034 (0.022)	0.221 (0.196)	-0.034 (-0.005)	0.034 (0.02)	-0.221 (-0.042)	0.221 (0.18)
	MAIAC	1 (10)	0.115 (0.08)	- (0.027)	- (0.764)	0.043 (0.036)	0.272 (0.323)	-0.043 (-0.032)	0.043 (0.032)	-0.272 (-0.285)	0.272 (0.286)
	FillSAT	1 (9)	0.121 (0.103)	- (0.021)	- (0.223)	0.037 (0.028)	0.236 (0.244)	-0.037 (-0.012)	0.037 (0.024)	-0.236 (-0.107)	0.236 (0.209)
	CMAQ	1 (10)	0.08 (0.062)	- (0.015)	- (0.731)	0.078 (0.052)	0.494 (0.464)	-0.078 (-0.049)	0.078 (0.049)	-0.494 (-0.441)	0.494 (0.441)
2/14/13	MISR	- (7)	- (0.105)	- (0.022)	- (0.953)	- (0.01)	- (0.089)	- (-0.007)	- (0.008)	- (-0.062)	- (0.074)
	MAIAC	1 (9)	0.169 (0.086)	- (0.045)	- (0.898)	0.018 (0.036)	0.122 (0.317)	0.018 (-0.027)	0.018 (0.032)	0.122 (-0.243)	0.122 (0.279)
	FillSAT	1 (7)	0.14 (0.105)	- (0.022)	- (0.953)	0.011 (0.012)	0.075 (0.104)	-0.011 (-0.009)	0.011 (0.01)	-0.075 (-0.081)	0.075 (0.084)
	CMAQ	1 (9)	0.118 (0.122)	- (0.021)	- (-0.478)	0.033 (0.039)	0.217 (0.345)	-0.033 (0.009)	0.033 (0.033)	-0.217 (0.078)	0.217 (0.289)

Pearson Correlation (R); Modeled results (MODEL); Surface Observations (SURF); Root Mean Square Error (RMSE)= $\sqrt{\frac{1}{N}\sum_N (MODEL - SURF)^2}$;

Mean Bias (MB) = $\frac{1}{N}\sum_N (MODEL - SURF)$; Mean Absolute Error (MAE) = $\frac{1}{N}\sum_N |MODEL - SURF|$; Normalized RMSE (NRMSE) = $\frac{\sqrt{\frac{1}{N}\sum_N (MODEL-SURF)^2}}{\frac{1}{N}\sum_N (SURF)}$; Normalized Mean Bias (NMB) = $\frac{\sum_N (MODEL-SURF)}{\sum_N (SURF)}$; Normalized Mean Absolute Error by obs. (NME) = $\frac{\sum_N |MODEL-SURF|}{\sum_N (SURF)}$.

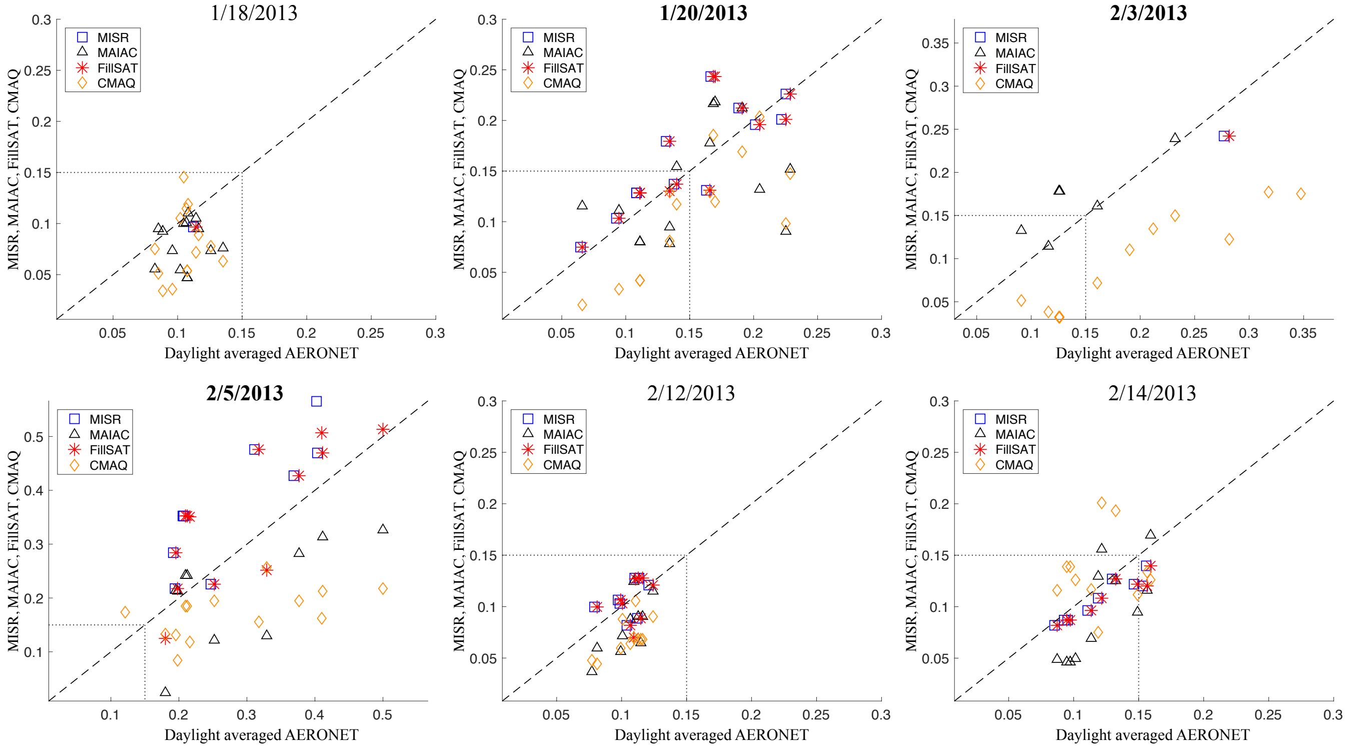

Figure S2: Snapshot vs. daylight-average AOD. Scatter plot comparison of MISR-RA, MAIAC, gap-filled MISR-RA (i.e., FillSAT) snapshots with AERONET and CMAQ daylight-averaged results. The MAIAC and AERONET AOD comparison is plotted at 550 nm, while the MISR-RA and AERONET AOD comparison is at 558 nm; the dotted line indicates the 0.15 AOD threshold; a 1:1 dashed line is shown for reference.

Table S5: Summary Statistics for AERONET Coincidences with all datasets using daylight average AOD≥0.15 (All AOD).

Date	Dataset	No. Coincident Observations	Mean	SD	Spatial R	RMSE	NRMSE	MB	MAE	NMB	NME
1/18/13	MISR	- (1)	- (0.097)	- (-)	- (-)	- (0.016)	- (0.141)	- (-0.016)	- (0.016)	- (-0.141)	- (0.141)
	MAIAC	- (14)	- (0.08)	- (0.022)	- (0.076)	- (0.036)	- (0.336)	- (-0.025)	- (0.028)	- (-0.24)	- (0.261)
	FillSAT	- (1)	- (0.097)	- (-)	- (-)	- (0.018)	- (0.156)	- (-0.018)	- (0.018)	- (-0.156)	- (0.156)
	CMAQ	- (14)	- (0.078)	- (0.033)	- (0.206)	- (0.043)	- (0.403)	- (-0.028)	- (0.037)	- (-0.265)	- (0.348)
1/20/13	MISR	6 (12)	0.202 (0.163)	0.039 (0.053)	0.326 (0.847)	0.036 (0.031)	0.188 (0.203)	0.007 (0.013)	0.027 (0.022)	0.038 (0.084)	0.137 (0.148)
	MAIAC	7 (14)	0.171 (0.137)	0.049 (0.052)	-0.785 (0.4)	0.07 (0.056)	0.363 (0.366)	-0.022 (-0.016)	0.059 (0.046)	-0.114 (-0.107)	0.305 (0.304)
	FillSAT	7 (14)	0.208 (0.167)	0.039 (0.054)	0.114 (0.81)	0.044 (0.034)	0.225 (0.222)	0.014 (0.014)	0.034 (0.025)	0.074 (0.088)	0.176 (0.16)
	CMAQ	7 (14)	0.151 (0.108)	0.038 (0.059)	-0.127 (0.776)	0.062 (0.057)	0.323 (0.375)	-0.043 (-0.045)	0.048 (0.047)	-0.221 (-0.293)	0.247 (0.309)
2/3/13	MISR	1 (1)	0.242 (0.242)	- (-)	- (-)	0.034 (0.034)	0.123 (0.123)	-0.034 (-0.034)	0.034 (0.034)	-0.123 (-0.123)	0.123 (0.123)
	MAIAC	2 (6)	0.2 (0.167)	0.055 (0.044)	- (0.85)	0.005 (0.035)	0.026 (0.247)	0.004 (0.026)	0.004 (0.026)	0.02 (0.181)	0.02 (0.184)
	FillSAT	1 (1)	0.242 (0.242)	- (-)	- (-)	0.039 (0.039)	0.139 (0.139)	-0.039 (-0.039)	0.039 (0.039)	-0.139 (-0.139)	0.139 (0.139)
	CMAQ	10 (14)	0.197 (0.152)	0.108 (0.117)	0.954 (0.965)	0.175 (0.154)	0.501 (0.545)	-0.152 (-0.13)	0.152 (0.13)	-0.435 (-0.462)	0.435 (0.462)
2/5/13	MISR	9 (9)	0.374 (0.374)	0.12 (0.12)	0.832 (0.832)	0.113 (0.113)	0.401 (0.401)	0.093 (0.093)	0.098 (0.098)	0.332 (0.332)	0.348 (0.348)
	MAIAC	10 (10)	0.211 (0.211)	0.095 (0.095)	0.617 (0.617)	0.115 (0.115)	0.402 (0.402)	-0.076 (-0.076)	0.095 (0.095)	-0.265 (-0.265)	0.33 (0.33)
	FillSAT	13 (13)	0.35 (0.35)	0.124 (0.124)	0.777 (0.777)	0.095 (0.095)	0.323 (0.323)	0.057 (0.057)	0.082 (0.082)	0.195 (0.195)	0.278 (0.278)
	CMAQ	13 (14)	0.172 (0.172)	0.047 (0.045)	0.603 (0.544)	0.146 (0.142)	0.5 (0.505)	-0.121 (-0.109)	0.121 (0.116)	-0.414 (-0.388)	0.414 (0.415)
2/12/13	MISR	- (8)	- (0.107)	- (0.017)	- (0.391)	- (0.016)	- (0.156)	- (0.003)	- (0.014)	- (0.025)	- (0.135)
	MAIAC	- (10)	- (0.08)	- (0.027)	- (0.752)	- (0.03)	- (0.289)	- (-0.024)	- (0.027)	- (-0.234)	- (0.263)
	FillSAT	- (10)	- (0.105)	- (0.021)	- (0.286)	- (0.02)	- (0.184)	- (-0.002)	- (0.017)	- (-0.021)	- (0.154)
	CMAQ	- (11)	- (0.07)	- (0.018)	- (0.668)	- (0.037)	- (0.352)	- (-0.034)	- (0.034)	- (-0.329)	- (0.329)
2/14/13	MISR	2 (9)	0.13 (0.108)	0.014 (0.021)	- (0.941)	0.026 (0.017)	0.17 (0.137)	-0.025 (-0.013)	0.025 (0.013)	-0.161 (-0.11)	0.161 (0.11)
	MAIAC	2 (11)	0.143 (0.096)	0.038 (0.046)	- (0.759)	0.029 (0.039)	0.186 (0.326)	-0.015 (-0.025)	0.025 (0.036)	-0.095 (-0.21)	0.161 (0.294)
	FillSAT	2 (9)	0.13 (0.108)	0.014 (0.021)	- (0.942)	0.029 (0.019)	0.185 (0.152)	-0.028 (-0.016)	0.028 (0.016)	-0.177 (-0.128)	0.177 (0.128)
	CMAQ	2 (11)	0.13 (0.134)	0.005 (0.036)	- (0.058)	0.028 (0.043)	0.178 (0.354)	-0.028 (0.013)	0.028 (0.038)	-0.176 (0.11)	0.176 (0.316)

*MISR, MAIAC, and FillSAT datasets are not daytime averages, but Terra overpass backfilled values.

Table S6: Summary of daytime or diurnal to Terra overpass hour ratios at AERONET locations for AOD≥0.15 (All AOD).

Date	Dataset	Ratio
1/18/13	AERONET (daytime-to-hr)	- (1.038)
	CMAQ (daytime-to-hr)	- (1.264)
	CMAQ (diurnal-to-hr)	- (1.152)
	FCMAQ (diurnal-to-hr)	- (1.376)
1/20/13	AERONET (daytime-to-hr)	0.928 (0.928)
	CMAQ (daytime-to-hr)	0.985 (1.159)
	CMAQ (diurnal-to-hr)	1.068 (1.158)
	FCMAQ (diurnal-to-hr)	1.052 (1.264)
2/3/13	AERONET (daytime-to-hr)	0.89 (0.864)
	CMAQ (daytime-to-hr)	1.037 (1.041)
	CMAQ (diurnal-to-hr)	1.034 (1.05)
	FCMAQ (diurnal-to-hr)	1.141 (1.153)
2/5/13	AERONET (daytime-to-hr)	0.945 (0.988)
	CMAQ (daytime-to-hr)	1.011 (1.015)
	CMAQ (diurnal-to-hr)	1.149 (1.143)
	FCMAQ (diurnal-to-hr)	1.082 (1.087)
2/12/13	AERONET (daytime-to-hr)	0.783 (0.95)
	CMAQ (daytime-to-hr)	0.978 (1.065)
	CMAQ (diurnal-to-hr)	1.123 (1.08)
	FCMAQ (diurnal-to-hr)	1.049 (1.161)
2/14/13	AERONET (daytime-to-hr)	1.053 (1.064)
	CMAQ (daytime-to-hr)	0.968 (0.91)
	CMAQ (diurnal-to-hr)	1.069 (0.992)
	FCMAQ (diurnal-to-hr)	1.04 (1.011)

Table S7: Statistical summary of comparison between AQS or CSN daily concentrations coincidences and each modeled or satellite derived dataset stratified by pollutant and day.

PM2.5_FRM

Date	Dataset	No. Coincident Observations	Mean	SD	Spatial R	RMSE	NRMSE	MB	MAE	NMB	NME
1/18/13	CMAQ	8	36.33	10.84	0.19	12.27	0.34	0.23	9.01	0.01	0.25
	FCMAQ	8	38.35	9.65	0.11	12.30	0.34	2.25	8.60	0.06	0.24
	FillSAT	8	29.33	5.25	0.89	8.56	0.24	-6.77	7.18	-0.19	0.20
	Opt	8	38.96	5.91	0.85	6.03	0.17	2.87	5.27	0.08	0.15
1/20/13	CMAQ	12	25.09	15.07	0.82	13.42	0.39	-9.36	10.76	-0.27	0.31
	FCMAQ	12	27.18	15.41	0.80	12.50	0.36	-7.27	10.59	-0.21	0.31
	FillSAT	12	23.19	14.23	0.86	14.28	0.41	-11.27	12.21	-0.33	0.35
	Opt	12	32.40	14.49	0.95	6.02	0.17	-2.06	4.57	-0.06	0.13
2/3/13	CMAQ	8	21.97	2.35	0.30	4.40	0.21	1.34	4.04	0.06	0.20
	FCMAQ	8	23.63	3.50	0.74	4.16	0.20	2.99	3.83	0.15	0.19
	FillSAT	8	23.09	9.32	0.72	6.85	0.33	2.46	5.26	0.12	0.26
	Opt	8	22.71	5.55	0.74	4.08	0.20	2.08	3.19	0.10	0.15
2/5/13	CMAQ	8	22.20	13.56	0.94	6.55	0.24	-4.74	4.76	-0.18	0.18
	FCMAQ	8	24.49	13.90	0.92	5.62	0.21	-2.44	4.35	-0.09	0.16
	FillSAT	8	38.55	27.23	0.59	23.75	0.88	11.62	16.70	0.43	0.62
	Opt	8	26.50	8.16	0.73	8.80	0.33	-0.43	6.10	-0.02	0.23
2/12/13	CMAQ	20	14.86	4.42	0.58	4.70	0.28	-1.89	3.73	-0.11	0.22
	FCMAQ	20	16.04	4.19	0.53	4.51	0.27	-0.71	3.46	-0.04	0.21
	FillSAT	20	11.82	3.72	0.19	7.45	0.44	-4.93	6.41	-0.29	0.38
	Opt	20	16.42	5.76	0.84	3.10	0.19	-0.33	2.38	-0.02	0.14
2/14/13	CMAQ	7	24.25	9.94	0.92	4.92	0.22	2.19	4.76	0.10	0.22
	FCMAQ	7	26.29	10.16	0.90	6.27	0.28	4.23	5.76	0.19	0.26
	FillSAT	7	11.50	5.34	0.88	12.58	0.57	-10.56	11.96	-0.48	0.54
	Opt	7	17.42	11.59	0.98	5.10	0.23	-4.64	4.64	-0.21	0.21

NH4

Date	Dataset	No. Coincident Observations	Mean	SD	Spatial R	RMSE	NRMSE	MB	MAE	NMB	NME
2/3/13	CMAQ	1	2.80	-	-	1.03	0.58	1.03	1.03	0.58	0.58
	FCMAQ	1	3.60	-	-	1.83	1.03	1.83	1.83	1.03	1.03
	FillSAT	1	2.58	-	-	0.81	0.46	0.81	0.81	0.46	0.46
	Opt	1	4.84	-	-	3.07	1.74	3.07	3.07	1.74	1.74
2/12/13	CMAQ	3	1.96	0.29	0.93	0.36	0.21	0.29	0.29	0.18	0.18
	FCMAQ	3	2.48	0.40	0.99	0.82	0.49	0.81	0.81	0.49	0.49
	FillSAT	3	1.48	0.50	-0.79	0.79	0.47	-0.19	0.66	-0.11	0.40
	Opt	3	3.61	2.15	1.00	2.37	1.42	1.94	1.94	1.16	1.16

Table S7: ***continued***

NO3

Date	Dataset	No. Coincident Observations	Mean	SD	Spatial R	RMSE	NRMSE	MB	MAE	NMB	NME
2/3/13	CMAQ	1	8.05	-	-	1.88	0.30	1.88	1.88	0.30	0.30
	FCMAQ	1	8.63	-	-	2.46	0.40	2.46	2.46	0.40	0.40
	FillSAT	1	7.41	-	-	1.24	0.20	1.24	1.24	0.20	0.20
	Opt	1	7.96	-	-	1.79	0.29	1.79	1.79	0.29	0.29
2/12/13	CMAQ	7	4.58	2.62	0.97	0.76	0.18	0.39	0.63	0.09	0.15
	FCMAQ	7	5.05	2.57	0.94	1.27	0.30	0.87	0.94	0.21	0.22
	FillSAT	7	3.32	2.11	0.69	2.11	0.50	-0.86	1.57	-0.21	0.37
	Opt	7	5.30	3.61	1.00	1.31	0.31	1.11	1.11	0.27	0.27

SO4

Date	Dataset	No. Coincident Observations	Mean	SD	Spatial R	RMSE	NRMSE	MB	MAE	NMB	NME
2/3/13	CMAQ	1	1.55	-	-	0.41	0.36	0.41	0.41	0.36	0.36
	FCMAQ	1	1.39	-	-	0.25	0.22	0.25	0.25	0.22	0.22
	FillSAT	1	1.43	-	-	0.29	0.26	0.29	0.29	0.26	0.26
	Opt	1	1.36	-	-	0.22	0.19	0.22	0.22	0.19	0.19
2/12/13	CMAQ	7	0.62	0.18	0.97	0.08	0.11	-0.05	0.05	-0.07	0.07
	FCMAQ	7	0.63	0.10	0.96	0.12	0.18	-0.04	0.09	-0.05	0.13
	FillSAT	7	0.56	0.25	-0.31	0.37	0.55	-0.11	0.29	-0.16	0.43
	Opt	7	0.72	0.24	0.75	0.16	0.24	0.05	0.10	0.08	0.15

EC

Date	Dataset	No. Coincident Observations	Mean	SD	Spatial R	RMSE	NRMSE	MB	MAE	NMB	NME
2/3/13	CMAQ	1	0.00	-	-	1.10	1.00	-1.10	1.10	-1.00	1.00
	FCMAQ	1	0.00	-	-	1.10	1.00	-1.10	1.10	-1.00	1.00
	FillSAT	1	0.13	-	-	0.97	0.88	-0.97	0.97	-0.88	0.88
	Opt	1	1.91	-	-	0.81	0.74	0.81	0.81	0.74	0.74
2/12/13	CMAQ	4	0.00	0.00	0.70	0.89	1.01	-0.88	0.88	-1.00	1.00
	FCMAQ	4	0.00	0.00	0.69	0.89	1.01	-0.88	0.88	-1.00	1.00
	FillSAT	4	0.00	0.00	0.64	0.88	1.00	-0.88	0.88	-1.00	1.00
	Opt	4	1.53	0.30	0.73	0.67	0.77	0.65	0.65	0.74	0.74

OC

Date	Dataset	No. Coincident Observations	Mean	SD	Spatial R	RMSE	NRMSE	MB	MAE	NMB	NME
2/3/13	CMAQ	1	6.00	-	-	0.30	0.05	0.30	0.30	0.05	0.05
	FCMAQ	1	5.25	-	-	0.45	0.08	-0.45	0.45	-0.08	0.08
	FillSAT	1	5.52	-	-	0.18	0.03	-0.18	0.18	-0.03	0.03
	Opt	1	4.77	-	-	0.93	0.16	-0.93	0.93	-0.16	0.16
2/12/13	CMAQ	4	4.58	0.97	0.77	0.99	0.26	0.80	0.82	0.21	0.22
	FCMAQ	4	4.30	0.63	0.76	0.78	0.21	0.52	0.65	0.14	0.17
	FillSAT	4	3.84	0.91	-0.68	1.54	0.41	0.06	1.46	0.02	0.39
	Opt	4	3.34	0.59	0.96	0.61	0.16	-0.44	0.51	-0.12	0.13

Table S8: Comparison of temporal R^2, spatial R^2, spatiotemporal R^2, mean bias, and root means square error values between surface observations and all simulation, including 10-fold 10% holdout cross-validation (10-WH CV), at all monitor locations and for 52 days.

Species	Dataset	Temporal R^2	Spatial R^2	Spatiotemporal R^2	Mean Bias	RMSE
$PM_{2.5}$	CMAQ	0.65	0.87	0.67	0.00	0.45
	FCMAQ	1.00	0.99	0.88	0.00	0.27
	OPT	1.00	0.96	0.87	-0.02	0.28
	FCMAQ 10-WH CV	0.95	0.68	0.80	0.24	0.32
	OPT 10-WH CV	0.95	0.69	0.79	0.24	0.33
NH4	CMAQ	0.70	0.67	0.61	0.29	1.69
	FCMAQ	1.00	0.99	0.95	0.71	0.81
	OPT	1.00	0.88	0.93	0.53	0.81
	FCMAQ 10-WH CV	-	0.98	1.00	1.88	1.02
	OPT 10-WH CV	-	0.98	1.00	1.86	1.01
SO4	CMAQ	0.29	0.22	0.36	0.00	0.51
	FCMAQ	0.99	0.98	0.94	0.05	0.19
	OPT	0.99	0.96	0.92	0.01	0.19
	FCMAQ 10-WH CV	0.98	0.74	0.79	0.11	0.23
	OPT 10-WH CV	0.98	0.73	0.78	0.11	0.23
NO3	CMAQ	0.69	0.77	0.79	0.13	0.67
	FCMAQ	1.00	0.99	0.99	0.28	0.40
	OPT	1.00	0.93	0.98	0.21	0.40
	FCMAQ 10-WH CV	-	0.79	0.88	0.40	0.63
	OPT 10-WH CV	-	0.79	0.88	0.42	0.65
OC	CMAQ	0.62	0.78	0.63	-0.10	0.31
	FCMAQ	1.00	0.99	0.98	-0.12	0.14
	OPT	1.00	0.95	0.97	-0.16	0.15
	FCMAQ 10-WH CV	-	0.98	0.75	-0.14	0.22
	OPT 10-WH CV	-	0.97	0.73	-0.13	0.23
EC	CMAQ	0.62	0.44	0.43	0.25	0.68
	FCMAQ	1.00	0.99	0.97	0.68	0.67
	OPT	1.00	0.98	0.97	0.65	0.65
	FCMAQ 10-WH CV	-	0.94	0.32	0.49	0.62
	OPT 10-WH CV	-	0.93	0.31	0.50	0.65

Pearson Squared Correlation (R^2); Modeled results (MODEL); Surface Observations (SURF); Covariance (cov); Standard Deviation (SD); monitor location(m_i); day observed (t); Number of monitors (N);

Temporal $R = \frac{1}{N}\sum_{i=1}^{N} \frac{cov\left(MODEL_{m_i,t}, SURF_{m_i,t}\right)}{SD_{MODEL_{m_i,t}} SD_{SURF_{m_i,t}}}$; Spatial $R = \frac{cov\left(\left(\frac{1}{N}\sum_N MODEL_m\right)_t, \left(\frac{1}{N}\sum_N SURF_m\right)_t\right)}{SD_{\left(\frac{1}{N}\sum_N MODEL_m\right)_t} SD_{\left(\frac{1}{N}\sum_N SURF_m\right)_t}}$;

Spatiotemporal $R = \frac{cov\left(MODEL_{m,t}, SURF_{m,t}\right)}{SD_{MODEL_{m,t}} SD_{SURF_{m,t}}}$;

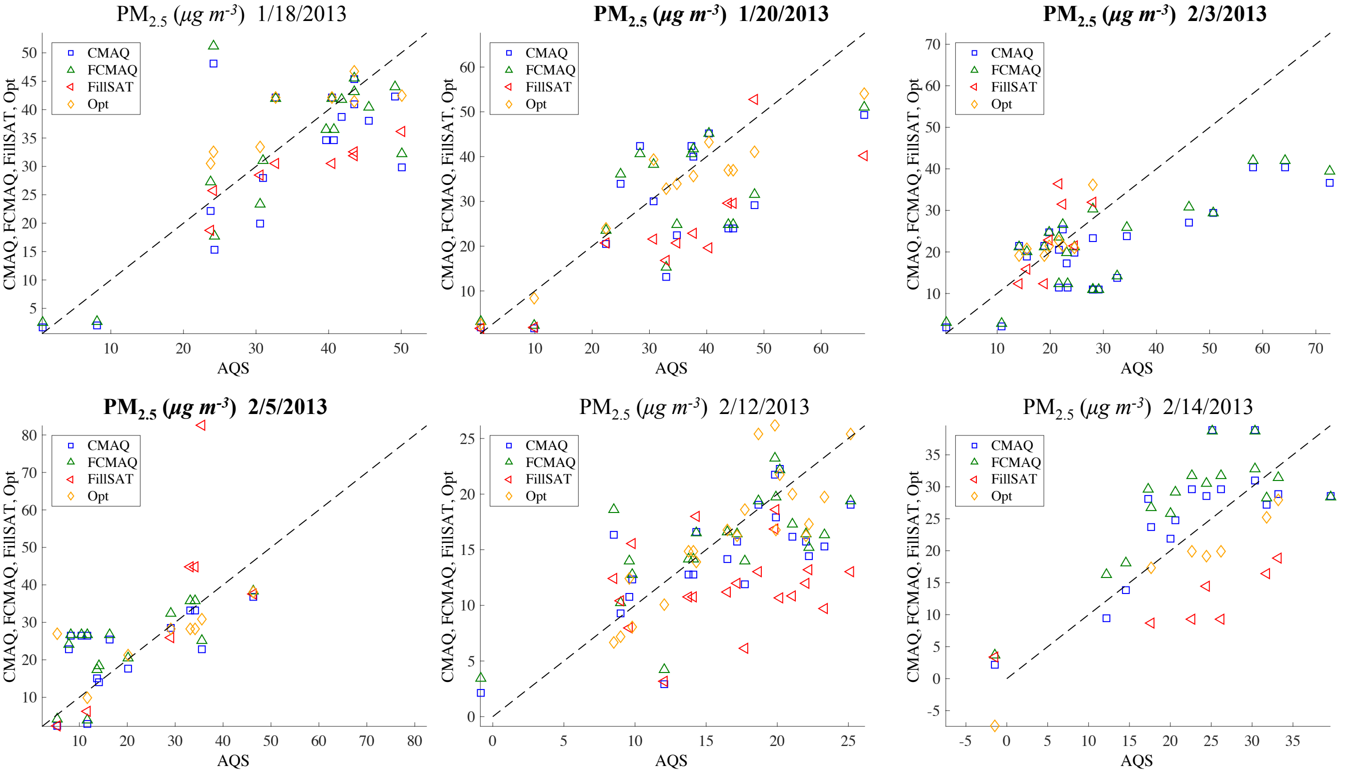

Figure S3: Scatterplot comparison of diurnally-averaged AQS $PM_{2.5}$ coincidences with CMAQ (i.e., model), FCMAQ (i.e., fused surface measurements + model), FillSAT, and Opt (i.e., optimized surface + satellite measurements + model) results are show for all days. The satellite datasets are derived from Terra overpass snapshots. A 1:1 dashed line is shown for reference.

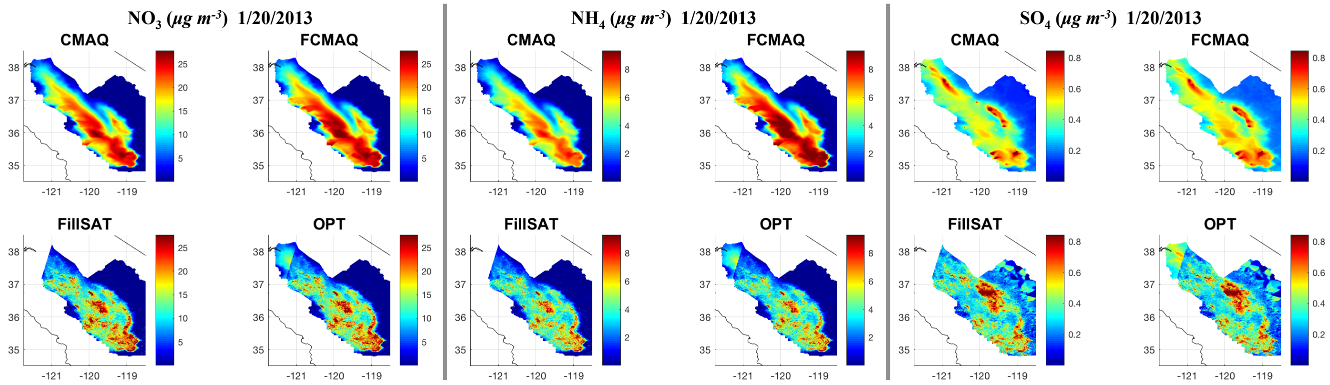

Figure S4: NH_4, SO_4, and NO_3 calculated concentration maps for January 20th, a day void of speciated ground-observations.

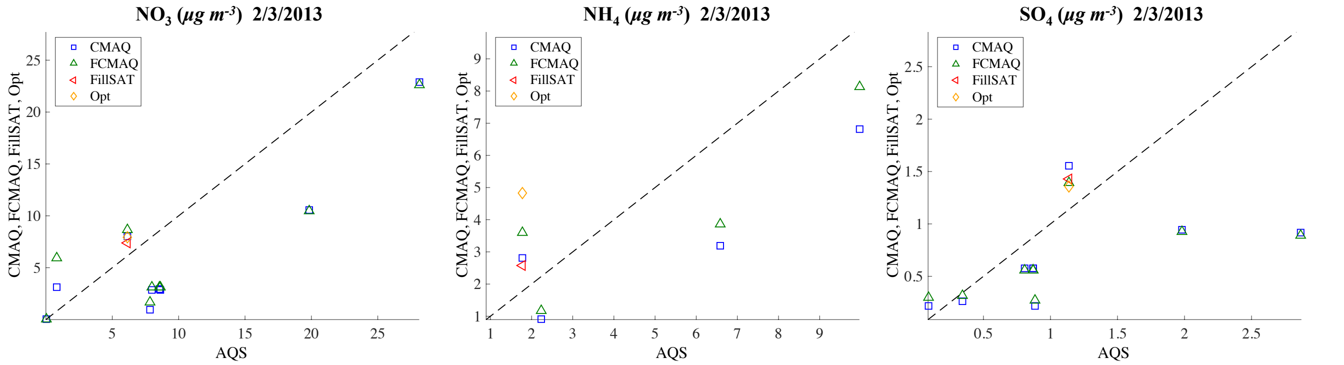

Figure S5: Scatterplot comparison of diurnally averaged AQS NO_3, NH_4, and SO_4 coincidences with CMAQ, FCMAQ, FillSAT, and Opt are show for January 3rd. The satellite datasets are derived from Terra overpass snapshots. A 1:1 dashed line is shown for reference.

References

Bond, T. C., and Bergstrom, R. W.: Light absorption by carbonaceous particles: An investigative review, Aerosol science and technology, 40, 27-67, 2006.

Chow, J. C., Lowenthal, D. H., Chen, L. W. A., Wang, X., and Watson, J. G.: Mass reconstruction methods for PM2.5: a review, Air Quality Atmosphere and Health, 8, 243-263, 2015.

Diner, D. J., Martonchik, J. V., Kahn, R. A., Pinty, B., Gobron, N., Nelson, D. L., and Holben, B. N.: Using angular and spectral shape similarity constraints to improve MISR aerosol and surface retrievals over land, Remote Sensing of Environment, 94, 155-171, 2005.

Friberg, M. D., Zhai, X., Holmes, H. A., Chang, H. H., Strickland, M. J., Sarnat, S. E., Tolbert, P. E., Russell, A. G., and Mulholland, J. A.: Method for fusing observational data and chemical transport model simulations to estimate spatiotemporally resolved ambient air pollution, Environmental science & technology, 50, 3695-3705, 2016.

Friberg, M. D., Kahn, R. A., Holmes, H. A., Chang, H. H., Sarnat, S. E., Tolbert, P. E., Russell, A. G., and Mulholland, J. A.: Daily ambient air pollution metrics for five cities: Evaluation of data-fusion-based estimates and uncertainties, Atmospheric Environment, 158, 36-50, 2017.

Kahn, R. A., and Gaitley, B. J.: An analysis of global aerosol type as retrieved by MISR, J. Geophys. Res. Atmos., 120, 4248-4281, 2015.

Limbacher, J. A., and Kahn, R. A.: MISR research-aerosol-algorithm refinements for dark water retrievals, Atmos. Meas. Tech., 7, 3989-4007, 2014.

Limbacher, J. A., and Kahn, R. A.: Updated MISR dark water research aerosol retrieval algorithm–Part 1: Coupled 1.1 km ocean surface chlorophyll a retrievals with empirical calibration corrections, Atmos. Meas. Tech., 10, 1539, 2017.

Malm, W. C., and Hand, J. L.: An examination of the physical and optical properties of aerosols collected in the IMPROVE program, Atmospheric Environment, 41, 3407-3427, 2007.

Malm, W. C., Schichtel, B. A., and Pitchford, M. L.: Uncertainties in PM2. 5 gravimetric and speciation measurements and what we can learn from them, Journal of the Air & Waste Management Association, 61, 1131-1149, 2011.

North, P. R. J., Briggs, S. A., Plummer, S. E., and Settle, J. J.: Retrieval of land surface bidirectional reflectance and aerosol opacity from ATSR-2 multiangle imagery, IEEE Transactions On Geoscience and Remote Sensing, 37, 526-537, 1999.

Park, R. S., Song, C. H., Han, K. M., Park, M. E., Lee, S. S., Kim, S. B., and Shimizu, A.: A study on the aerosol optical properties over East Asia using a combination of CMAQ-simulated aerosol optical properties and remote-sensing data via a data assimilation technique, Atmos. Chem. Phys., 11, 12275-12296, 2011.

Song, C. H., Park, M. E., Lee, K. H., Ahn, H. J., Lee, Y., Kim, J. Y., Han, K. M., Kim, J., Ghim, Y. S., and Kim, Y. J.: An investigation into seasonal and regional aerosol characteristics in East Asia using model-predicted and remotely-sensed aerosol properties, Atmos. Chem. Phys., 8, 6627-6654, 2008.

Sun, H., Biedermann, L., and Bond, T. C.: Color of brown carbon: A model for ultraviolet and visible light absorption by organic carbon aerosol, Geophys. Res. Lett., 34, 2007.

Tegen, I., Hollrig, P., Chin, M., Fung, I., Jacob, D., and Penner, J.: Contribution of different aerosol species to the global aerosol extinction optical thickness: Estimates from model results, Journal of Geophysical Research: Atmospheres, 102, 23895-23915, 1997.

Zamora, I., and Jacobson, M.: Measuring and modeling the hygroscopic growth of two humic substances in mixed aerosol particles of atmospheric relevance, Atmos. Chem. Phys., 13, 8973-8989, 2013.
